# Steroids and/or Non-Steroidal Anti-Inflammatory Drugs as Postoperative Treatment after Trabeculectomy—12-Month Results of a Randomized Controlled Trial

**DOI:** 10.3390/jcm13030887

**Published:** 2024-02-02

**Authors:** Afrouz Ahmadzadeh, Line Kessel, Bo Simmendefeldt Schmidt, Miriam Kolko, Daniella Bach-Holm

**Affiliations:** 1 Department of Ophthalmology, Copenhagen University Hospital—Rigshospitalet, 2100 Copenhagen, Denmark; 2 Department of Clinical Medicine, University of Copenhagen, 2200 Copenhagen, Denmark; 3 Department of Physics, Technical University of Denmark, 2800 Kongens Lyngby, Denmark; 4 Department of Drug Design and Pharmacology, University of Copenhagen, 2200 Copenhagen, Denmark

**Keywords:** glaucoma, intraocular pressure, NSAID, steroid, trabeculectomy

## Abstract

This prospective randomized controlled trial aimed to compare the efficacy and safety of topical preservative-free diclofenac (DICLO) to dexamethasone (DEX) eyedrops, and their combination (DEX+DICLO) after trabeculectomy. Sixty-nine patients with medically uncontrolled glaucoma were randomized to receive topical postoperative treatment with DICLO (*n* = 23), DEX (*n* = 23), or a combination of DEX and DICLO (*n* = 23). The primary outcome was the intraocular pressure (IOP) 12 months postoperatively. Secondary outcomes included surgical success, failure, visual field, and visual acuity from baseline to 12 months postoperatively. IOP reached the lowest point one day after trabeculectomy. At 12 months, IOP was 10.0 mmHg (95% CI, 8.4–11.6 mmHg) for DICLO, 10.9 mmHg (95% CI, 9.4–12.3 mmHg) for DEX, and 11.2 mmHg (95% CI, 9.1–13.3 mmHg) for DEX+DICLO. There were no significant differences in IOP, surgical success, failure, visual field, or visual acuity between the DICLO, DEX, or DEX+DICLO groups. We found that topical diclofenac was not statistically different from topical dexamethasone in controlling IOP 12 months after trabeculectomy. Combining diclofenac and dexamethasone offered no added IOP control compared to dexamethasone alone.

## 1. Introduction

Trabeculectomy is the gold standard for medically uncontrolled glaucoma [1]. The procedure enables direct aqueous outflow from the anterior chamber of the eye into the subconjunctival space, thereby reducing IOP [2,3]. Unlike most surgical procedures, the effectiveness and optimal filtration rate of a trabeculectomy depends on inhibiting wound healing and controlling the amount of postoperative inflammation that can lead to tissue fibrosis.

Anti-inflammatory prophylaxis following trabeculectomy is paramount in balancing inflammation and tissue fibrosis, and topical steroids are very often used postoperatively to facilitate this balance [4,5,6]. A well-documented side effect of steroid treatment is the development or progression of cataract, regardless of whether it is administered topically or systemically [7,8,9,10]. Furthermore, some patients may experience increased IOP (steroid response) during treatment, despite a functioning bleb, giving the impression that the filtration surgery has failed [11,12,13,14].

Non-steroidal anti-inflammatory drugs (NSAIDs) may be considered an alternative, as they are not associated with cataract formation or IOP elevation, and have been comparable to topical steroids in managing inflammation after trabeculectomy and cataract surgery in previous small studies [15,16,17,18]. NSAIDs also have the added benefit of providing postoperative pain relief, although some patients may complain about stinging when applied [15,16,17].

We conducted the Steroids and/or Non-steroidal Anti-inflammatory Drugs in the Postoperative Regime After Trabeculectomy (SNAP) Study, a randomized controlled clinical trial to evaluate the efficacy and safety of topical and preservative-free anti-inflammatory prophylactic treatments, including dexamethasone, diclofenac, or a combination of these regimens.

## 2. Materials and Methods

### 2.1. Study Design

This prospective randomized controlled trial was conducted at the Department of Ophthalmology at Rigshospitalet-Glostrup, Denmark. Before recruitment was initiated, the study was approved by the Danish Medicines Agency (Journal nr.: 2018082465), Ethical committee (Journal nr.: H-18056701), and the Danish Data Protection Agency (VD-2018-477, I-Suite nr.: 6736), and registered at the European Union Drug Regulating Authorities Clinical Trials Database (EudraCT, 2018-001855-10, 10 Oktober 2018) and http://www.clinicaltrials.gov (accessed on 12 December 2023)/(NCT04054830). At the time of enrollment, participants provided written informed consent and received no compensation or incentives for participation in the trial. The trial was monitored by an independent body, conducted in accordance with Good Clinical Practice guidelines, and adhered to the tenets of the Declaration of Helsinki [19]. The Consolidated Standards of Reporting Trials (CONSORT) reporting guidelines were followed for all reporting aspects.

### 2.2. Intervention

Participants were randomized to one of three interventional groups comparing anti-inflammatory regimes after trabeculectomy. Preservative-free diclofenac (Voltaren Ophtha 1 mg/mL, GSK Consumer Healthcare) (DICLO) or a combination of preservative-free dexamethasone (Monopex 1 mg/mL, Théa) and preservative-free diclofenac (Voltaren Ophtha 1 mg/mL, GSK Consumer Healthcare) (DEX+DICLO) was compared with the control group receiving preservative-free dexamethasone (Monopex 1 mg/mL, Théa) (DEX).

A topical antibiotic (Chloramphenicol 5 mg/mL) was prescribed four times daily for the first week. Anti-inflammatory prophylaxis was scheduled to last a minimum of nine weeks, administered six times daily for the first two weeks, followed by four times daily for the remaining four weeks. Thus, a total of 12 drops were used daily in the DEX+DICLO group in the first two weeks. Depending on the clinical condition of the eye (anterior chamber flare, conjunctival injection, SUN grading, and central corneal thickness), the anti-inflammatory prophylaxis was reduced by one daily drop per week from week 6. The topical treatment was changed to preservative-free DEX if anti-inflammatory medication was necessary for more than 15 weeks after the filtering surgery.

Trial medications were prepared by Glostrup Apotek, Denmark, following good manufacturing practices.

### 2.3. Participants

Participants were included from 1 August 2019 to 11 July 2021. Patients older than 50 years with primary open-angle glaucoma (POAG), pseudoexfoliation glaucoma (PEXG), pigment dispersion glaucoma (PDS), or ocular hypertension (OHT) with medically uncontrolled IOP were eligible for the study. Exclusion criteria included pre-menopausal women, prior intraocular surgery other than cataract surgery, a medical history of anterior segment dysgenesis, uveitic/inflammatory glaucoma, angle closure glaucoma, traumatic glaucoma, or neovascular glaucoma. Patients receiving systemic NSAIDs or steroids, known steroid responders, or who had any allergies to pharmaceuticals used in the study were also excluded. Participants were required to comply with study procedures and provide informed consent for participation. Only one eye of each eligible participant was included in the study.

### 2.4. Randomization and Masking

Before study initiation, an independent researcher generated a 123-long block-randomized list using https://www.sealedenvelope.com/simple-randomiser/v1/lists (accessed on 12 December 2023) and uploaded it to the randomization instrument Research Electronic Data Capture (REDCap) [20,21]. Enrolled participants were randomized to one of three intervention groups using a computerized 1:1:1 algorithm. If both eyes were eligible, the algorithm would decide which eye to include [20,21]. Due to the mono- or combination therapy study design and the absence of a vehicle-only study arm, participants could not be masked to treatment status, but the primary outcome assessors were masked to randomization status. All statistical calculations were performed in a masked manner.

### 2.5. Surgical Technique

Three experienced surgeons performed all trabeculectomies. A limbal peritomy (fornix-based) was made superiorly, the conjunctiva was undermined, and a standard limbus-based scleral flap, 3 × 4 mm with 2/3 of scleral thickness, was dissected. A sponge soaked in 0.2 mg/mL Mitomycin (MMC) was applied subconjunctivally for 3 min, followed by irrigation of the area with balanced salt solution. Two sutures 10-0 ethilon (ETHICON, Johnson & Johnson SURGICAL TECHNOLOGIES, USA) were placed in the corners of the scleral flap followed by a sclerostomy and peripheral iridectomy. The flap was sutured to limit outflow and the conjunctiva was closed with corneal single sutures (10-0 ethilon) at the end of the procedure, ensuring water tightness. The procedure was completed by injecting 1 mL cefuroxime 2.5 mg/mL into the anterior chamber and injecting 0.5 mL of 4 mg/mL dexamethasone subconjunctivally 180° away from the trabeculectomy. According to the surgeon’s preference, the trabeculectomies were performed using peribulbar or topical anesthesia except for one procedure conducted under general anesthesia.

### 2.6. Follow-Up Examinations

Participants were examined at baseline and day 1, week 1, 2, 3, 4, 6, and month 3, 6, and 12 after the operation. A time frame for postoperative visits was prespecified, ±2 days for the weekly visits, ±7 days for the 3- and 6-month visits, and ±1 month for the 12-month visit. The examinations included IOP assessed using Goldmann applanation tonometry [22]. Visual field mean defect was obtained with the dG30 program of Octopus 900 (Haag-Streit, Switzerland) and analyzed with EyeSuite Perimetry viewer. Distance best-corrected visual acuity (BCVA) logarithm of the minimum angle of resolution (logMAR) was examined by using the Early Treatment of Diabetic Retinopathy Study (ETDRS) chart [23]. Adverse events were grouped into early- (occurring within the first three months) and late-onset. Bleb leaks were classified into two categories: bleb leak–slit lamp intervention, which involved managing the bleb leak using a scleral lens, and bleb leak–surgical intervention, which referred to re-suturing the bleb in the operating room.

### 2.7. Outcomes

The primary outcome measure was the IOP 12 months postoperatively. Secondary outcome measures were surgical success and failure rates, visual field mean defect, BCVA, and adverse events from baseline to 12 months postoperatively.

A target pressure was set for each participant based on their glaucoma diagnosis and the degree of their visual field defect, as used in other studies [24,25]:

- Ocular hypertension, target < 25 mmHg;
- MD (mean defect) < 6 dB, target < 21 mmHg;
- MD 6 dB–12 dB, target < 18 mmHg;
- MD > 12 dB, target < 15 mmHg.

Surgical success and failure criteria were defined according to the achievement of target pressure without the use of medication (complete success) or with the use of glaucoma medication (qualified success) [22]. An IOP ≤ 5 mmHg after 4 weeks, or the need for subsequent glaucoma surgery (e.g., revision of the trabeculectomy, aqueous shunt surgery, or photocoagulation of ciliary body) was considered failure. Laser suture lysis and needling were not defined as subsequent glaucoma surgery or failures.

### 2.8. Sample Size Calculations

The primary outcome of the study was IOP 12 months following surgery. The sample size calculation was based on patients undergoing trabeculectomy at the department of Ophthalmology, Rigshospitalet-Glostrup. We expected a postoperative IOP of 12.1 mmHg (SD 3.9) 12 months after surgery based on our own data. Using a sampling of 1.1:1, a power of 0.8, and a type 1 error of 0.05, a minimum of 16 participants was required in each treatment group to detect a difference in mean IOP at 12 months between the groups, assuming a SD of 3.9 mmHg. A total of 72 participants were enrolled in the study to compensate for possible dropouts.

### 2.9. Statistical Analysis

The statistical software R, version 4.1.2, in RStudio, version 2022.02.3 build 492, and the LMMstar package were used for the analyses (R Program for Statistical Computing) [26]. The Shapiro–Wilk test and the visual inspection of QQ-plots revealed that the data for IOP, visual field, and visual acuity were normally distributed at all time points.

A constrained linear mixed model with the same unstructured covariance pattern for all groups with inherent baseline adjustment was applied for pairwise comparisons of all time values across the three treatment groups. The model includes time and the interaction between treatment and time as fixed effects to account for correlation between repeated measurements and potential variance changes over time. Missing data were addressed with maximum likelihood estimation, which provides unbiased estimates of treatment effects and time when data are missing at random (MAR). Using a best-case/worst-case scenario, sensitivity analysis was conducted by substituting 10th and 90th percentiles of the observed data for the missing data. The cumulative rate of glaucoma reoperation was compared with the Kaplan–Meier survival analysis test.

ETDRS chart measurements were converted to the logarithm of the minimum angle of resolution (logMAR) equivalents for the visual acuity data analysis.

The IOP analysis was adjusted for multiple testing using Bonferroni correction, resulting in a significance level of 0.0125. The secondary outcomes were corrected for multiple testing with the Benjamini–Hochberg procedure, which controls the false discovery rate (FDR). An adjusted *p*-value < 0.05 was considered statistically significant. Included participants provided data until exclusion.

## 3. Results

**Figure 1:**
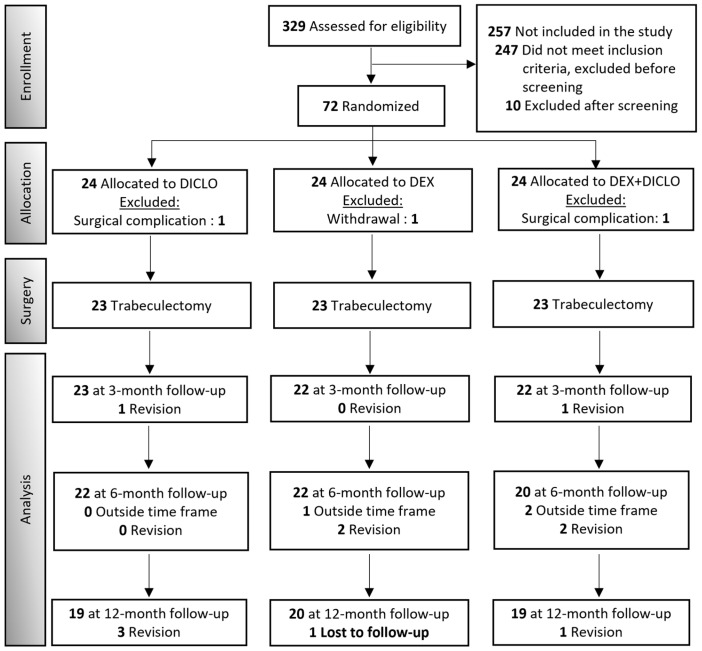
CONSORT diagram for the primary outcome. The participants who were examined outside the ± one-week window for the 6-month visit returned to the 12-month visit inside the ± one-month frame. The need for a revision of the trabeculectomy resulted in exclusion from the trial.

Eighty-two patients were assessed for eligibility between August 2019 and June 2021. Seventy-two patients were included and randomized evenly in the intervention groups. One participant in the DEX group withdrew consent. Two participants (DICLO: *n* = 1, DEX+DICLO: *n* = 1) had complications during surgery and were excluded from the study. Overall, a total of 69 eyes of 69 patients (30 women [43%]; 39 men [57%]), with a mean age of 71.3 ± 9.0 years (range, 51 to 88 years) provided baseline data; see Table 1 for baseline characteristics. Fifty-eight participants completed the one-year follow-up; see Figure 1 for a CONSORT flow diagram.

### 3.1. Intraocular Pressure

**Figure 2:**
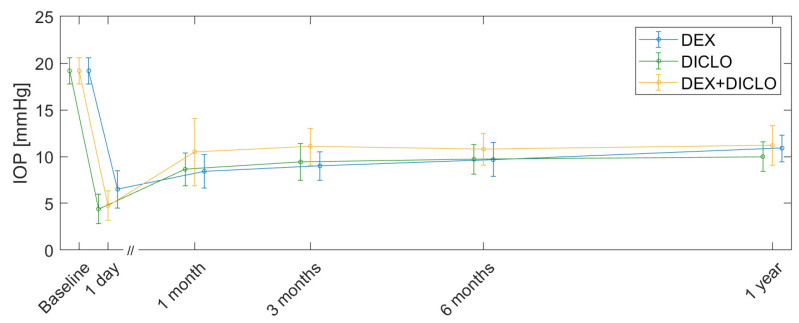
Change in IOP from baseline to 12 months after trabeculectomy. Error bars represent mean and 95% confidence intervals. See Figure 1, CONSORT diagram, for the number of eyes at each timepoint. For the change in IOP from baseline to 3 months, see Figure 2 [11].

IOP reached a minimum value one day after trabeculectomy. From a baseline mean of 19.2 mmHg (95% CI, 17.8–20.6 mmHg), the pressure decreased to 4.4 mmHg (95% CI, 2.8–6.0 mmHg) for DICLO, 6.5 mmHg (95% CI, 4.5–8.5 mmHg) for DEX, and 4.8 mmHg (95% CI, 3.2–6.3 mmHg) for DEX+DICLO. The IOP gradually increased with time, but all postoperative IOP measurements were significantly lower than preoperative values (*p* < 0.05). At 12 months, IOP remained significantly reduced to 10.0 mmHg (95% CI, 8.4–11.6 mmHg) for DICLO, 10.9 mmHg (95% CI, 9.4–12.3 mmHg) for DEX, and 11.2 mmHg (95% CI, 9.1–13.3 mmHg) for DEX+DICLO. There were no significant differences in IOP at any time point between the three intervention groups (Table 2 and Table S1, Figure 2).

### 3.2. Surgical Success and Probability of Success

Table 3 reports the success rates in the three intervention groups. At 12 months, the probability of complete success was 69.1% for all participants, 69.6% for DICLO, 72.7% for DEX, and 65.5% for DEX+DICLO. The qualified success rate was 11.8% for all participants, 13.0% for DICLO, 13.6% for DEX, and 8.8% for DEX+DICLO. The overall failure rate was 19.1% for all participants, 17.4% for DICLO, 13.6% for DEX, and 26.1% for DEX+DICLO. No statistical differences were identified for the success or failure rates among the three groups (*p* = 0.55).

**Figure 3:**
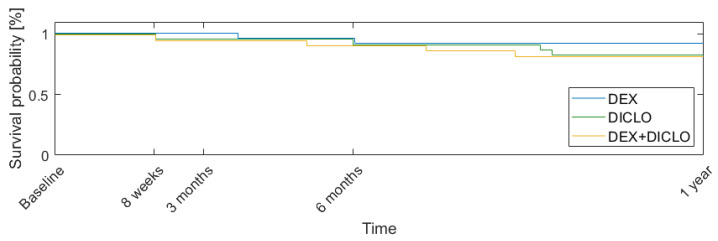
Kaplan–Meier plot showing the probability of success for the first year following trabeculectomy.

The cumulative probabilities of success at 12 months for the trabeculectomy was 82.6% for both the DICLO and DEX+DICLO groups, and 91.3% for the DEX group (logrank test with *p*-value = 0.6). The success curves are presented in Figure 3.

### 3.3. Visual Field

The changes in visual fields during the study period are reported in Table 2 and supplemental Figure 4. The mean defect at baseline was 15.3 dB (95% CI, 13.9–16.7 dB). Twelve months postoperatively, the mean visual field defect was 15.7 dB (95% CI, 13.2–18.3 dB) for DICLO, 16.0 dB (95% CI, 12.9–19.2 µm) for DEX, and 14.4 dB (95% CI, 11.8–17.0 dB) for DEX+DICLO. No statistically significant differences between the groups were found at any time point, and importantly, no statistically significant visual field progression was found in any of the groups.

### 3.4. Visual Acuity

**Figure 5:**
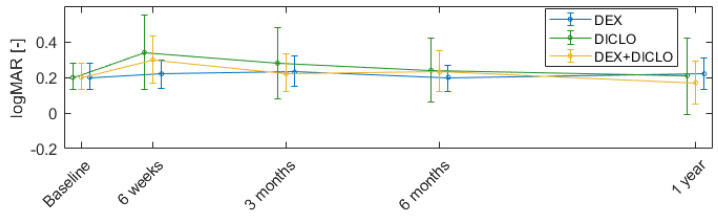
Change in visual acuity from baseline to 12 months after trabeculectomy. Error bars represent mean and 95% confidence intervals.

Most patients experienced a dip in visual acuity around six weeks after surgery, but best corrected distance visual acuity was back to baseline and stable in all groups from 3 to 12 months postoperatively; see Table 2. The postoperative visual acuity of the three interventional groups did not differ significantly from the preoperative value. See Figure 5 for an illustration of the development over time. Two participants allocated to DEX+DICLO experienced vision deterioration from cataract that required surgery.

### 3.5. Adverse Events and Additional Treatment

During the first twelve months postoperatively, none of the participants required additional anti-inflammatory prophylaxis beyond the initial nine weeks. Late postoperative adverse events affected 24.6% (*n* = 17) of the participants (Table 4). The most common complication was needling, followed by hypotony. A total of 10 participants had an unfavorable IOP that could not be controlled with medication or needling and required additional glaucoma surgery during the first year postoperatively (failures) due to posterior fibrosis, hypotony, or bleb leak. The participants who had a flattened bleb were not the same as the ones who underwent needling. Needling was not included in the failure criteria. There were no significant differences in adverse events between the three interventional groups.

## 4. Discussion

This randomized clinical trial demonstrates that the choice of using topical diclofenac (DICLO), dexamethasone (DEX), or a combination of the two (DEX+DICLO) as anti-inflammatory prophylaxis following trabeculectomy does not influence the IOP, surgical success, failure, progression of visual field mean defect, or visual acuity from baseline to 12 months postoperatively. No statistically significant differences were found between the treatment groups. Prophylactic anti-inflammatory treatment with DICLO is non-inferior to DEX in controlling IOP up to 12 months following trabeculectomy. The combination treatment with DEX+DICLO showed no added value compared to DEX monotherapy. Importantly, none of the participants required additional anti-inflammatory treatment following trabeculectomy.

Regarding the rationale for examining the 12-month results, it is crucial to recognize that trabeculectomy efficacy depends on inhibiting wound healing and controlling postoperative inflammation, which can lead to tissue fibrosis. Monitoring long term IOP serves as an indicator of trabeculectomy success. Despite participants discontinuing anti-inflammatory treatments after nine weeks, the 12-month evaluation enables us to assess the sustained impact on IOP and trabeculectomy success rates. We found that the postoperative IOP was significantly lower than the preoperative value, and the IOP-lowering effect of the trabeculectomy was comparable between the interventional groups at every visit. The intriguing finding of this study is that the IOP-lowering effect after trabeculectomy did not differ significantly between the anti-inflammatory prophylactic alternatives. This finding is consistent with a previous study that showed that use of prednisolone or DICLO eyedrops after trabeculectomy resulted in similar IOP-lowering effect 6 months postoperatively [17]. In addition, a meta-analysis including participants who underwent phacotrabeculectomy or trabeculectomy concluded that in terms of outcome and risks around 6 months after surgery, NSAID eyedrops were non-inferior when used alone or in combination with topical steroids in patients undergoing trabeculectomy [18]. The comparable anti-inflammatory efficiency of NSAIDs and steroids after glaucoma procedures has been investigated with varying outcomes. As previously described, Kent et al. found comparable IOP using either DICLO or steroids following trabeculectomy. Breusegem et al. [27] investigated the beneficial effects of preoperative prophylaxis with topical NSAIDs, steroids, or artificial tears. They found that prescribing steroids or NSAIDs decreased the need for needling after trabeculectomy, while the steroid group also received less postoperative IOP-lowering medication than the two other groups [27].

Glaucoma therapy aims to preserve visual function and prevent further glaucomatous optic nerve damage. IOP is currently the only risk factor amenable to therapeutic intervention. A definition of success based solely on IOP does not account for the damaging effect on the optic nerve head, as tolerance levels vary between individuals. We defined the success criteria according to the preoperative severity of visual field defects and the achievement of target pressure without the use of medication (complete success) or with the use of glaucoma medication (qualified success). No significant difference in complete or qualified success was seen between the treatment groups. Likewise, the failure rates of not achieving target pressure or requiring additional surgery were comparable across the three treatment groups.

The majority of participants had a decline in visual acuity six weeks following surgery but visual acuity returned to the same preoperative level within 3 months. We also discovered that the visual field defects were stable and unaltered throughout the 1 year study period.

It has been demonstrated in randomized clinical trials that lowering IOP delays the progression of visual field loss [28,29,30,31,32]. However, visual field defects often worsen despite surgery. Approximately a third of eyes undergoing trabeculectomy continue to progress five years later [33]. We did not observe significant changes in mean defect from baseline to 12 months after trabeculectomy in any of the interventional groups. The level of IOP reduction after trabeculectomy is important for visual field deterioration postoperatively [33], and a lower IOP is associated with a slower progression of visual field defects [28]. Others have reported a continued deterioration of visual fields following trabeculectomy, although at a slower rate [34], whereas the level of IOP reduction was not correlated to the rate of visual field loss [34,35,36,37]. This is likely explained by individual variations in susceptibility to IOP. This is why we chose our success criteria to depend on the extent of preoperative visual field damage.

The optimal filtration rate after trabeculectomy depends on controlling the amount of postoperative inflammation, which, if not carried out appropriately, may lead to tissue fibrosis. The use of topical anti-inflammatory prophylaxis is an essential component of this regulation, and most often steroids are used [4,5]. However, steroids can induce elevated IOP, cataract formation, or an increased risk of infection [6,38]. Frequent transient side effects of topical NSAIDs include redness, irritation, and impaired vision. NSAID-induced corneal melt (NICM) is a rare side effect that mainly affects individuals with corneal impairment due to systemic immune diseases, diabetes, or ocular surgery [39,40]. The topically administered NSAID serves as a catalyst and transforms the epithelial defect into a melt [40]. In the literature, NICM has been variously reported. Although it has been described in case reports [39,40], when investigating the safety and effectiveness of NSAID eyedrops after cataract surgery, no cases of NICM were detected in a retrospective cohort study of over 16,070 eyes [41] or in two randomized controlled trials [42,43]. The duration of treatment varied between 14 days and to the surgeon’s preference. The risk of NICM may be higher after trabeculectomy due to prolonged treatment. In our study, neither corneal thinning nor any statistically significant differences in adverse events were seen between treatment groups [11]. This finding is analogous to a phacotrabeculectomy study in which NSAIDs were administered for nine weeks postoperatively [44].

The strengths of this study include the large sample size, a randomized design, masked primary outcome assessors, and that the study was monitored by the GCP unit in accordance with GCP quality standards. Only one participant was lost to follow-up due to a medical challenge unrelated to the eye disorder; thus, the study has a low dropout rate. The study results were reported in accordance with the World Glaucoma Association Guidelines [22], contributing to greater uniformity in the field of glaucoma. Due to one group receiving two types of eyedrops, the study could not be fully masked. However, the primary outcome assessors were masked to the randomization status, and all statistical analyses were performed in a masked manner. Younger patients undergoing trabeculectomy are at a higher risk of bleb fibrosis, and this factor should be considered when interpreting our study outcomes. The rationale for not considering needling as a failure criterion or a definition of qualified success is based on the intent to distinguish between interventions that are part of the standard postoperative management and those that indicate a more significant challenge or failure of the procedure. Needling is a common and planned intervention to address issues like bleb fibrosis or excessive scarring that can occur in the postoperative period. By not categorizing needling as a failure criterion, this study aims to differentiate between routine management procedures and instances where more substantial interventions are needed due to the failure of the trabeculectomy. Needling, in this context, is seen as a part of the anticipated postoperative care rather than an indication of failure. As noted, there was no significant difference in the number of needling interventions between the three interventional groups. The standard postoperative care for trabeculectomy in our setting includes the administration of subconjunctival dexamethasone at the conclusion of the surgery. However, this practice is not universal, and its use introduces a steroidal effect even in the group that received only diclofenac. This could potentially limit the generalizability of our findings and preclude readers from considering non-steroidal treatment options as a complete replacement for topical steroids.

## 5. Conclusions

This study demonstrates that postoperative treatment with diclofenac eyedrops compared with conventional dexamethasone drops showed no statistically significant difference in IOP control 12 months after trabeculectomy, and that combining diclofenac and dexamethasone offered no added advantage. All three postoperative treatments were associated with stabilizing visual fields. Our findings indicate that diclofenac may be considered a viable alternative to dexamethasone for postoperative treatment following trabeculectomy, but studies with a longer follow-up are required.

## Figures and Tables

**Figure 4:**
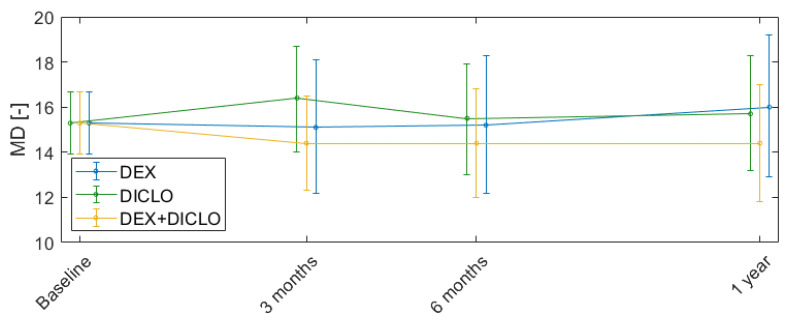
Change in mean defect from baseline to 12 months after trabeculectomy. Error bars represent mean and 95% confidence intervals.

**Table 1:** Baseline characteristics.

	All Participants	DICLO	DEX	DEX+DICLO	*p*-Value
Participants, *n*	69	23	23	23	
Female/Male, *n* (%)	30/39 (43/57)	7/16 (30/70)	12/11 (52/48)	11/12 (48/52)	0.29
Age (yrs), mean (SD)	71.3 (9.0)	69.3 (7.2)	73.7 (8.2)	71.0 (11.0)	0.25
Glaucoma diagnoses					0.39
*HTG, n (%)*	55 (79.7)	17 (73.9)	17 (73.9)	21 (91.3)	
*NTG, n (%)*	5 (7.2)	2 (8.7)	2 (8.7)	1 (4.4)	
*OHT, n (%)*	1 (1.4)	0 (0.0)	1 (4.4)	0 (0.0)	
*PDG, n (%)*	2 (2.9)	1 (4.4)	0 (0.0)	1 (4.4)	
*PEXG, n (%)*	6 (8.7)	3 (13.0)	3 (13.0)	0 (0.0)	
No. of glaucoma medication, mean (SD)	3.6 (0.9)	3.7 (0.9)	3.4 (0.9)	3.7 (0.8)	0.40
*Prostaglandin, n (%)*	69 (100)	23 (100)	23 (100)	23 (100)	
*Beta-blocker, n (%)*	64 (92.8)	21 (91.3)	21 (91.3)	22 (95.7)	
*Alpha agonist, n (%)*	43 (62.3)	16 (69.6)	12 (52.2)	15 (65.2)	
*Topical Carbonic Anhydrase Inhibitor, n (%)*	62 (90.0)	21 (91.3)	20 (87.0)	21 (91.3)	
*Systemic Carbonic Anhydrase Inhibitor, n (%)*	10 (14.5)	3 (13.0)	2 (8.7)	5 (21.7)	
IOP (mmHg), mean (SD)	19.1 (5.9)	18.4 (6.4)	18.4 (5.7)	20.4 (5.8)	0.48
MD (dB), mean (SD)	15.3 (5.8)	15.8 (5.7)	15.2 (6.4)	14.9 (5.4)	0.89
Visual acuity (logMAR)	0.20 (0.3)	0.27 (0.44)	0.19 (0.23)	0.15 (0.22)	0.45
Phakia/Pseudophakia, *n* (%)	46/23 (67/33)	18/5 (78/22)	11/12 (48/52)	17/6 (74/26)	0.06

Abbreviations: HTG = high tension glaucoma; NTG = normal tension glaucoma; OHT = ocular hypertension; PDG = pigment dispersion glaucoma; PEXG = pseudoexfoliative glaucoma; IOP = intraocular pressure; MD = mean defect; logMAR = logarithm to the minimal angle of resolution.

**Table 2:** Postoperative results on IOP, visual field, and visual acuity.

	DICLO	DEX	DEX+DICLO
IOP *† [mmHg], mean (CI)			
Baseline	19.2 (17.8, 20.6)	19.2 (17.8, 20.6)	19.2 (17.8, 20.6)
1 d postop	4.4 (2.8, 6.0)	6.5 (5.0, 8.5)	4.8 (3.2, 6.3)
1 w postop	5.3 (3.9, 6.7)	5.8 (4.0, 7.6)	5.0 (3.8, 6.3)
2 w postop	6.1 (5.0, 7.2)	6.4 (4.7, 8.0)	6.8 (5.9, 7.8)
3 w postop	7.8 (6.5, 9.1)	7.8 (5.9, 9.8)	8.3 (7.0, 9.7)
4 w postop	8.7 (6.9, 10.4)	8.4 (6.7, 10.2)	10.5 (6.9, 14.1)
6 w postop	11.5 (8.8, 14.1)	10.5 (8.3, 12.6)	12.7 (10.4, 15.0)
3 m postop	9.4 (7.5, 11.4)	9.0 (7.5, 10.5)	11.1 (9.1, 13.0)
6 m postop	9.7 (8.2, 11.3)	9.7 (7.9, 11.5)	10.8 (9.1, 12.5)
12 m postop	10.0 (8.4, 11.6)	10.9 (9.4, 12.3)	11.2 (9.1, 13.3)
Change relative to control, mean (CI)	0.85 (−1.2, 2.9)	Control	−0.36 (−2.9, 2.1)
*p* value/adj-*p* value ^x^, 12 m	0.409/0.771		0.771/0.771
Visual field, MD*† [dB], mean (CI)			
Baseline	15.3 (13.9, 16.7)	15.3 (13.9, 16.7)	15.3 (13.9, 16.7)
3 m postop	16.4 (14.0, 18.7)	15.1 (12.2, 18.1)	14.4 (12.3, 16.5)
6 m postop	15.5 (13.0, 17.9)	15.2 (12.2, 18.3)	14.4 (12.0, 16.8)
12 m postop	15.7 (13.2, 18.3)	16.0 (12.9, 19.2)	14.4 (11.8, 17.0)
Change relative to control, mean (CI)	0.30 (−3.6, 4.2)	Control	1.62 (−2.3, 5.6)
*p* value/adj-*p* value ^x^, 12 m	0.878/0.878		0.413/0.826
Visual acuity *,† (logMAR), mean (CI)			
Baseline	0.20 (0.13, 0.28)	0.20 (0.13, 0.28)	0.20 (0.13, 0.28)
6 w postop	0.34 (0.13, 0.55)	0.22 (0.14, 0.30)	0.30 (0.17, 0.43)
3 m postop	0.28 (0.08, 0.48)	0.23 (0.15, 0.32)	0.22 (0.12, 0.33)
6 m postop	0.24 (0.06, 0.42)	0.20 (0.12, 0.27)	0.23 (0.12, 0.35)
12 m postop	0.21 (−0.01, 0.42)	0.22 (0.13, 0.31)	0.17 (0.05, 0.29)
Change relative to control, mean (CI)	0.01 (−0.21, 0.24)	Control	0.05 (−0.09, 0.19)
*p* value/adj-*p* value ^x^, 12 m	0.887/0.887		0.491/0.887

IOP = intra ocular pressure; postop = postoperatively; w = week; m = month; MD = mean defect, * Estimates were derived from the constrained linear mixed model with baseline adjustment. Changes in the DICLO and DEX+DICLO groups are presented as differences from the DEX group as mean (95% CI), † 95% CI. ^x^ Adjusted for false discovery rate.

**Table 3:** Complete and qualified surgical success and failure rates.

	All Participants	DICLO	DEX	DEX+DICLO
Participants, *n*	68	23	22 *	23
Complete success ^a^ *n* (%)	47 (69.1)	16 (69.6)	16 (72.7)	15 (65.2)
Qualified success ^b^ *n* (%)	8 (11.8)	3 (13.0)	3 (13.6)	2 (8.7)
*No. of glaucoma medication, mean (SD)*	1.9 (1.1)	1.3 (0.6)	2.7 (1.5)	1.5 (0.7)
Failure ^c^				
*Not achieving target IOP with medications ^d^ n (%)*	3 (4.4)	0 (0.0)	1 (4.5)	2 (8.7)
*Need for subsequent glaucoma surgery n (%)*	10 (14.7)	4 (17.4)	2 (9.1)	4 (17.4)

* one participant lost to follow-up; ^a^, achievement of target pressure without the use of medication; ^b^, achievement of target pressure with the use of medication; ^c^, not achieving target pressure with medications or need for subsequent glaucoma surgery; ^d^, participants scheduled for surgery or participants not interested in further surgical intervention.

**Table 4:** Adverse events and the need for added treatment.

	All Participants	DICLO	DEX	DEX+DICLO
Participants allocated to treatment, *n*	69	23	23	23
Participants excluded (failures), *n* (%)	10 (14.5)	4 (17.4)	2 (8.7)	4 (17.4)
*Cause of revision*				
*Hypotony*	2 (2.9)	1 (4.3)	1 (4.3)	0 (0.0)
*Flattened bleb*	6 (8.7)	3 (13.0)	1 (4.3)	2 (8.7)
*Bleb leak*	2 (2.9)	0 (0.0)	0 (0.0)	2 (8.7)
Early adverse events, *n* (%)				
Total *	43 (62.3)	14 (60.9)	14 (60.9)	15 (65.2)
*Hypotony +*	20 (29.0)	7 (30.4)	6 (26.1)	7 (30.4)
*Elevated IOP* ‡	8 (11.6)	4 (17.4)	2 (8.7)	2 (8.7)
*Bleb leak–slit lamp intervention*	13 (18.8)	4 (17.4)	6 (26.1)	3 (13.0)
*Bleb leak–surgical intervention*	5 (7.2)	1 (4.3)	2 (8.7)	2 (8.7)
*Corneal edema*	10 (14.5)	2 (8.7)	3 (13.0)	5 (21.7)
*Dryness*	4 (5.8)	0 (0.0)	2 (8.7)	2 (8.7)
*Corneal abrasion*	6 (8.7)	2 (8.7)	0 (0.0)	4 (17.4)
*Corneal dellen*	1 (1.4)	0 (0.0)	0 (0.0)	1 (4.3)
*Hyphema*	1 (1.4)	0 (0.0)	1 (4.3)	0 (0.0)
Late adverse events, *n* (%)				
Total *	17 (24.6)	5 (21.7)	5 (21.7)	7 (30.4)
*Hypotony*	2 (2.9)	1 (4.3)	1 (4.3)	0 (0.0)
*Diplopia*	1 (1.4)	0 (0.0)	0 (0.0)	1 (4.3)
*Needling*	7 (10.1)	3 (13.0)	1 (4.3)	3 (13.0)

* Total adverse events refer to the number of participants with one or more adverse events. +: managed with atropine due to choroidal detachment; ‡: managed with pressure-lowering medication or needling. Bleb leak–slit lamp intervention: managed with a scleral lens; Bleb leak–surgical intervention: re-suturing.

## Data Availability

The datasets generated during and/or analyzed during the current study are available from the corresponding author on reasonable request.

## Supplementary Materials

The following supporting information can be downloaded at: https://www.mdpi.com/article/10.3390/jcm13030887/s1, Table S1: Postoperative results on IOP with *p* values.
