# Supplementary material for: Steroids and/or Non-Steroidal Anti-Inflammatory Drugs as Postoperative Treatment after Trabeculectomy—12-Month Results of a Randomized Controlled Trial
Source: J Clin Med. 2024 Feb 2;13(3):887. doi: 10.3390/jcm13030887 (PMC10856597; doi:10.3390/jcm13030887)
Supplement: Supplementary file 1 [file jcm-13-00887-s001.zip › jcm-2829905-supplementary.pdf]

Table S1. Postoperative results on IOP with *p* values.			
	**DICLO**	**DEX**	**DEX+DICLO**
IOP*† [mmHg], mean (CI), *p* value⁺			
Baseline	19.2 (17.8, 20.6)	19.2 (17.8, 20.6)	19.2 (17.8, 20.6)
1 d postop	4.4 (2.8, 6.0); 0.076	6.5 (5.0, 8.5)	4.8 (3.2, 6.3); 0.799
1 w postop	5.3 (3.9, 6.7); 0.635	5.8 (4.0, 7.6)	5.0 (3.8, 6.3); 0.772
2 w postop	6.1 (5.0, 7.2); 0.747	6.4 (4.7, 8.0)	6.8 (5.9, 7.8); 0.507
3 w postop	7.8 (6.5, 9.1); 0.941	7.8 (5.9, 9.8)	8.3 (7.0, 9.7); 0.737
4 w postop	8.7 (6.9, 10.4); 0.879	8.4 (6.7, 10.2)	10.5 (6.9, 14.1); 0.255
6 w postop	11.5 (8.8, 14.1); 0.609	10.5 (8.3, 12.6)	12.7 (10.4, 15.0); 0.215
3 m postop	9.4 (7.5, 11.4); 0.638	9.0 (7.5, 10.5)	11.1 (9.1, 13.0); 0.118
6 m postop	9.7 (8.2, 11.3); 0.568	9.7 (7.9, 11.5)	10.8 (9.1, 12.5); 0.252
12 m postop	10.0 (8.4, 11.6); 0.955	10.9 (9.4, 12.3)	11.2 (9.1, 13.3); 0.291
Change relative to control, mean (CI)	0.85 (-1.2, 2.9)	Control	-0.36 (-2.9, 2.1)
P value/adj-*p* value ˣ, 12 m	0.409/0.771		0.771/0.771

IOP = intra ocular pressure; postop = postoperatively; w = week; m = month; *Estimates were derived from the constrained linear mixed model with baseline adjustment. Changes in the DICLO and DEX+DICLO groups are presented as differences from the DEX group as mean (95% CI), † 95% CI. ˣ Adjusted for false discovery rate. ⁺ *p* values presented indicate differences in IOP at any time point between the three intervention groups
